# Lumican Inhibits Osteoclastogenesis and Bone Resorption by Suppressing Akt Activity

**DOI:** 10.3390/ijms22094717

**Published:** 2021-04-29

**Authors:** Jin-Young Lee, Da-Ae Kim, Eun-Young Kim, Eun-Ju Chang, So-Jeong Park, Beom-Jun Kim

**Affiliations:** 1 Asan Institute for Life Sciences, Asan Medical Center, University of Ulsan College of Medicine, Seoul 05505, Korea; takewlsdud@gmail.com (J.-Y.L.); sizzzeri@gmail.com (D.-A.K.); 2 Department of Biomedical Sciences, Asan Medical Center, University of Ulsan College of Medicine, Seoul 05505, Korea; ssainokim76@gmail.com (E.-Y.K.); ejchang@amc.seoul.kr (E.-J.C.); 3 Division of Endocrinology and Metabolism, Asan Medical Center, University of Ulsan College of Medicine, Seoul 05505, Korea

**Keywords:** lumican, osteoclast, bone resorption, Akt signaling

## Abstract

Lumican, a ubiquitously expressed small leucine-rich proteoglycan, has been utilized in diverse biological functions. Recent experiments demonstrated that lumican stimulates preosteoblast viability and differentiation, leading to bone formation. To further understand the role of lumican in bone metabolism, we investigated its effects on osteoclast biology. Lumican inhibited both osteoclast differentiation and in vitro bone resorption in a dose-dependent manner. Consistent with this, lumican markedly decreased the expression of osteoclastogenesis markers. Moreover, the migration and fusion of preosteoclasts and the resorptive activity per osteoclast were significantly reduced in the presence of lumican, indicating that this protein affects most stages of osteoclastogenesis. Among RANKL-dependent pathways, lumican inhibited Akt but not MAP kinases such as JNK, p38, and ERK. Importantly, co-treatment with an Akt activator almost completely reversed the effect of lumican on osteoclast differentiation. Taken together, our findings revealed that lumican inhibits osteoclastogenesis by suppressing Akt activity. Thus, lumican plays an osteoprotective role by simultaneously increasing bone formation and decreasing bone resorption, suggesting that it represents a dual-action therapeutic target for osteoporosis.

## 1. Introduction

The small leucine-rich proteoglycans (SLRPs) are biologically active components of the extracellular matrix (ECM). SLRPs have relatively small (36–42 kDa) protein cores with leucine-rich repeat (LRR) motifs covalently linked to glycosaminoglycan (GAG) side chains [1,2]. The SLRP family has 18 members divided into five distinct classes based on chromosomal organization and functional and structural properties [1,3]. SLRPs directly or indirectly regulate multiple cellular processes through interactions with various growth factors, cytokines, cell surface receptors, and other ECM components [4]. Importantly, most SLRPs are enriched in skeletal tissues [5], and several lines of evidence indicate that specific SLRPs play critical roles in bone homeostasis [6,7]. For example, mice lacking biglycan, a class I SLRP, exhibit osteoporotic phenotypes caused by a reduction in new bone formation and an increase in osteoclastogenesis [8,9]. Deletion of epiphycan, a class III SLRP, causes shortening of the femur during growth, indicating that it has an important function in bone homeostasis [10].

The ubiquitously expressed protein lumican, originally identified as a major component of the cornea [11], has been utilized in a wide range of biological functions including cell proliferation, differentiation, migration, and adhesion, in a variety of tissues [12,13]. Although lumican belongs to the SLRP family and may therefore be involved in bone metabolism [5], the effects of lumican on bone biology are not yet fully understood. In vitro and animal experiments have demonstrated that lumican promotes preosteoblast viability and differentiation via integrin α2β1 and downstream ERK signaling, leading to bone formation [14]. To further elucidate the role of lumican in bone metabolism, we investigated its effects on osteoclasts and found that it inhibits osteoclastogenesis and bone resorption by suppressing Akt signaling.

## 2. Results

### 2.1. Inhibition of In Vitro Bone Resorption and Osteoclastogenesis by Lumican Treatment

**Figure 1:**
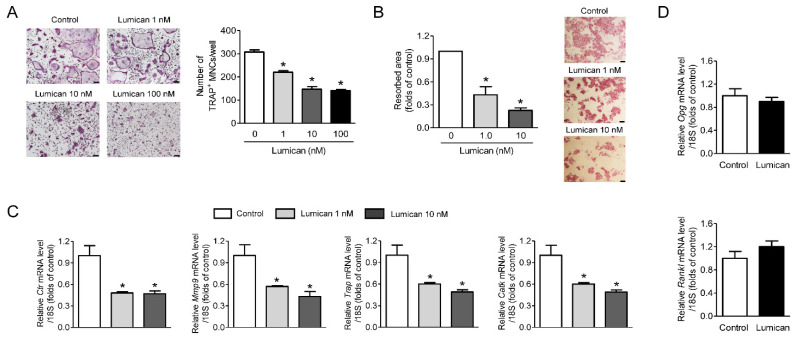
Lumican suppresses osteoclast differentiation and in vitro bone resorption. (**A**) Primary mouse BMMs were cultured for 4 days with 30 ng/mL RANKL, 30 ng/mL M-CSF, and the indicated concentrations of lumican. After the cells were stained with TRAP, TRAP-positive multinucleated cells (MNCs) (≥3 nuclei/cell) were counted to assess osteoclast differentiation (*n* = 5). (**B**) Mouse BMMs were cultured on dentin slices for 10 days in the presence of 30 ng/mL RANKL, 30 ng/mL M-CSF, and 1 nM or 10 nM lumican and stained with hematoxylin to visualize pit formation (*n* = 3). (**C**) Quantitative RT-PCR analysis of osteoclast differentiation markers in BMMs exposed to 30 ng/mL RANKL, 30 ng/mL M-CSF, and 1 or 10 nM lumican for 4 days (*n* = 4). (**D**) Quantitative RT-PCR analysis to measure *Opg* and *Rankl* expression in mouse calvaria osteoblasts exposed for 7 days to 10 mM β-glycerophosphate and 50 mg/mL ascorbic acid, with or without 10 nM lumican (*n* = 3). Scale bars: 100 μm (**A**) and 50 μm (**B**). Data are means ± SEM. * *p* < 0.05 vs. untreated control.

We treated primary mouse bone marrow macrophages (BMMs) with various concentrations of lumican in the presence of RANKL and M-CSF. Lumican inhibited osteoclast differentiation in a dose-dependent manner, as confirmed by Tartrate-resistant acid phosphatase (TRAP) staining, and the maximum effect was observed at 10 nM lumican (Figure 1A). In addition, 1 or 10 nM lumican decreased in vitro bone resorption relative to untreated control by 57% and 77%, respectively (Figure 1B). Consistent with this, the expression of osteoclastogenesis markers such as *Ctr*, *Mmp9*, *Trap*, and *CatK* was markedly decreased by lumican treatment (Figure 1C). Next, we investigated whether the potential effects of lumican on osteoclasts were mediated by modulation of OPG and RANKL production from osteoblasts. However, recombinant lumican did not affect the expression of *Opg* and *Rankl* in primary mouse calvaria osteoblasts (Figure 1D).

### 2.2. Lumican Decreases Bone Resorptive Activity as Well as Migration and Fusion of Preosteoclasts

**Figure 2:**
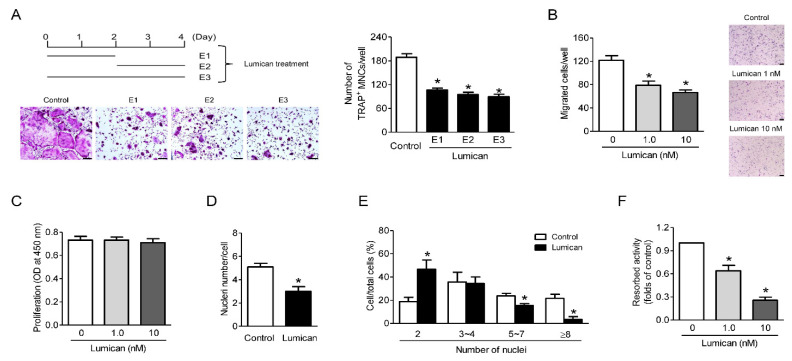
Lumican inhibits bone resorptive activity as well as migration and fusion of preosteoclasts. (**A**) Primary mouse BMMs were exposed to 10 nM lumican on the first 2 days (E1), last 2 days (E2), or entire 4 days (E3) of a 4-day culture in the presence of 30 ng/mL RANKL and 30 ng/mL M-CSF. After the cells were stained with TRAP, the number of TRAP-positive multinucleated cells (MNCs) (≥3 nuclei/cell) was determined to assess osteoclast differentiation (*n* = 3). (**B**) Mouse BMMs were incubated with 30 ng/mL M-CSF for 3 days, and then directional migration was assessed through a Boyden chamber system after treatment with 1 nM or 10 nM lumican for an additional 6 h (*n* = 5). (**C**) Mouse BMMs were cultured with 30 ng/mL M-CSF for 3 days, and then proliferation was assessed by BrdU assay after treatment with 1 or 10 nM lumican for an additional 1 day (*n* = 5). (**D**,**E**) TRAP staining of mouse BMMs was performed after treatment for 3 days with 30 ng/mL RANKL and 30 ng/mL M-CSF in the presence of 10 nM lumican (*n* = 3). (**D**) Nuclei per TRAP-positive cell. (**E**) Percentages of cells with the indicated numbers of nuclei. (**F**) After full differentiation of primary osteoclast precursors into osteoclasts by incubation for 3 days with 30 ng/mL RANKL and 30 ng/mL M-CSF, cells were seeded on dentine discs and cultured for an additional 3 days in the presence of 1 or 10 nM lumican with RANKL and M-CSF. Resorption pits were visualized by hematoxylin staining (*n* = 3). Scale bars: 100 μm (**A**) and 50 μm (**B**). Data are means ± SEM. * *p* < 0.05 vs. untreated control.

Osteoclastogenesis proceeds via the migration and proliferation of preosteoclasts, their fusion, and subsequent osteoclast differentiation, culminating in bone resorption [15,16]. When lumican was administered for a 2 day period (early: first 2 days; late: second 2 days) during 4 day cultures of primary mouse BMMs with RANKL and M-CSF, it inhibited both early and late stages of osteoclastogenesis (Figure 2A). Migration (Figure 2B), but not proliferation (Figure 2C), of preosteoclasts was suppressed by lumican. The number of nuclei per cell and the percentage of multinucleated cells was markedly reduced in the presence of lumican, indicating that lumican impaired preosteoclast fusion (Figure 2D,E, respectively). Lumican also decreased the resorptive activity per osteoclast (Figure 2F). Collectively, these findings indicate that lumican affects most stages of osteoclastogenesis in vitro.

### 2.3. Akt Signaling Mediates the Effects of Lumican on Osteoclastogenesis

**Figure 3:**
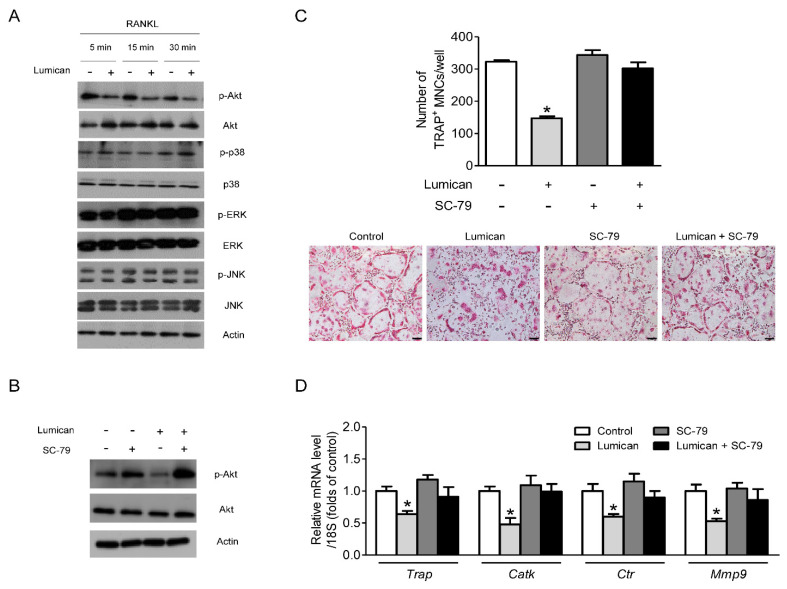
Suppression of the Akt signal mediates the effects of lumican on osteoclastogenesis. (**A**) Western blot to assess RANKL-dependent phosphorylation of downstream kinases in mouse BMMs after exposure to 10 nM lumican and 30 ng/mL RANKL following culture for 3 days in the presence of 30 ng/mL M-CSF (*n* = 5). (**B**) Western blot analysis to assess the phosphorylation of Akt in mouse BMMs after exposure to 30 ng/mL RANKL, 10 nM lumican, and 2 μg/mL SC-79 (Akt activator) following culture for 3 days in the presence of 30 ng/mL M-CSF (*n* = 3). (**C**,**D**) Primary mouse BMMs were co-treated with 30 ng/mL RANKL, 30 ng/mL M-CSF, 10 nM lumican, and 2 μg/mL SC-79 for 4 days (*n* = 4). (**C**) After the cells were stained with TRAP, the number of TRAP-positive multinucleated cells (MNCs) (≥ 3 nuclei/cell) was determined to assess osteoclast differentiation. (**D**) Quantitative RT-PCR analysis was performed to determine the levels of osteoclast differentiation markers. Scale bars: 100 μm (**C**). Data are means ± SEM. * *p* < 0.05 vs. untreated control.

To elucidate the action mechanism of lumican on osteoclasts, we focused on RANKL-dependent signaling, a critical pathway related to osteoclast differentiation [17]. Lumican inhibited the activity of Akt, but not MAP kinases such as p38, ERK, and JNK (Figure 3A). Importantly, co-treatment with the Akt activator SC-79 (Figure 3B) almost completely eliminated the effects of lumican on osteoclastogenesis (Figure 3C). Consistent with this, SC-79 attenuated the lumican-mediated reduction in expression of osteoclast differentiation markers (Figure 3D). Together, these data indicate that Akt plays a critical role in the effect of lumican on osteoclast biology.

### 2.4. Lumican Increases Apoptosis of Osteoclasts and Decreases Actin Ring Formation

**Figure 4:**
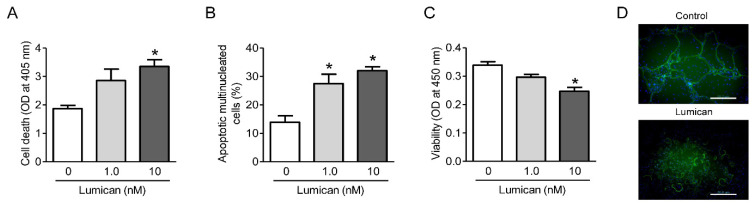
Lumican increases osteoclast apoptosis and decreases osteoclast viability and actin ring formation. (**A**,**B**) Primary mouse BMMs were cultured with 30 ng/mL RANKL and 30 ng/mL M-CSF for 4 days, and then RANKL was withdrawn and the cells were cultured with 1 nM or 10 nM lumican for an additional 1 day. Apoptosis was determined by Cell Death ELISA and TUNEL assays, respectively (*n* = 5). (**C**) Primary mouse BMMs were cultured with 30 ng/mL RANKL and 30 ng/mL M-CSF for 4 days, and then assayed for viability using a CCK-8 assay after treatment with 1 nM or 10 nM lumican for an additional 1 day (*n* = 3). (**D**) F-actin staining was performed to assess actin ring formation after treatment of mouse BMMs for 4 days with 30 ng/mL RANKL and 30 ng/mL M-CSF in the presence of 10 nM lumican (*n* = 3). Scale bars: 20 μm (**D**). Data are means ± SEM. * *p* < 0.05 vs. untreated control.

Akt is involved in the survival and microtubule stabilization in osteoclast lineages [18,19]. Hence, we investigated the effects of lumican on these processes. Cell death ELISA and TUNEL assays confirmed that lumican increased osteoclast apoptosis (Figure 4A,B, respectively) and the CCK-8 assays revealed that lumican decreased osteoclast viability (Figure 4C). In addition, actin ring formation was inhibited following treatment with lumican (Figure 4D). These results further support the Akt-mediated role of lumican on osteoclast lineages.

### 2.5. Lumican Stimulates the Polarization of M0 Macrophages to M1 Macrophages

**Figure 5:**
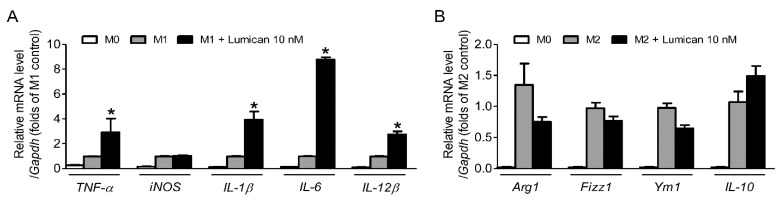
The effects of lumican on M1 or M2 polarization. (**A,B**) Primary mouse BMMs were incubated with 30 ng/mL M-CSF for 24 h and then treated with medium containing 10 ng/mL LPS and 10 ng/mL IFN-γ (M1 polarization) or 10 ng/mL IL-4 (M2 polarization) for an additional 24 h. Cells were assayed by quantitative RT-PCR analysis using (**A**) *TNF-α*, *iNOS*, *IL-1β*, *IL-6*, or *IL-12β* (M1 marker genes)-specific primers and (**B**) *Arg1*, *Fizz1*, *Ym1*, or *IL-10* (M2 marker genes)-specific primers (*n* = 3). PCR primer sequences were retrieved from the online PrimerBank database. Data are means ± SEM. * *p* < 0.05 vs. M1 control.

Akt activation is known to switch M0 macrophages to M2 macrophages rather than to M1 macrophages [20] and thus we evaluated the effects of lumican on M1 and M2 polarization to further clarify the role of Akt signaling in lumican-mediated changes induced in the osteoclast lineages. Recombinant lumican treatment significantly increased the mRNA expression of M1 markers including TNF-α, IL-1β, IL-6, and IL-12β (Figure 5A), while the mRNA expression of various M2 markers including Arg1, Fizz1, and Ym1 tended to decrease in the presence of lumican (Figure 5B). These results support our hypothesis that lumican inhibits Akt signaling in osteoclast lineages. Furthermore, considering that macrophages and osteoclasts are the two competing differentiation outcomes, from myeloid progenitors [21], the activation to M1 macrophages may partially explain the lumican-induced decrease in osteoclastogenesis.

## 3. Discussion

Osteoporosis, a common metabolic bone disease in older adults, is caused by an imbalance between osteoclastic breakdown and osteoblastic rebuilding during bone remodeling. Currently, anti-resorptives such as bisphosphate and denosumab are widely used for osteoporosis treatment. However, due to the temporal and spatial coordination of bone resorption with bone formation [22], these agents concomitantly suppress bone formation, limiting their therapeutic efficacy and raising concerns about their long-term adverse effects [23,24,25]. Accordingly, many researchers have sought to develop anti-osteoporotic drugs that can dissociate bone resorption from bone formation. Recent work showed that lumican exerts anabolic effects on bones by stimulating osteoblastogenesis [14]. Furthermore, this study revealed that lumican directly suppresses in vitro bone resorption as well as osteoclastogenesis. Taken together, these results suggest that lumican plays an osteoprotective role by simultaneously increasing bone formation and reducing bone resorption, and thus represents a potential dual-action therapeutic target for osteoporosis.

As a candidate receptor of lumican in osteoclasts, we focused on transmembrane integrin α2β1, which is involved in the activities of lumican in several cell types [26,27]. Recent work demonstrated that integrin α2β1 mediates the beneficial effects of lumican in osteoblasts [14]. Integrin αVβ3 was also regarded as a potential target because this is the most abundant form of integrins in osteoclasts [28]. However, treatment of osteoclasts with the integrin α2β1 inhibitor TC-I 15 or integrin αVβ3 inhibitor echistatin did not reverse the suppression of osteoclastogenesis by lumican treatment (Supplemental Figure S1A,B, respectively), suggesting that these are not the major receptors for lumican in osteoclasts. This unanswered question should be addressed by future experiments.

The inhibitory effects of lumican ranged from the early to the late stages of osteoclastogenesis. Hence, we initially investigated changes in RANKL-dependent signaling [17,29], which could account for the diverse roles of lumican on osteoclast biology. Western blot analyses revealed that lumican reduced the phosphorylation of Akt, but not JNK, p38, or ERK, whereas an Akt activator significantly attenuated the lumican-induced decrease in osteoclast differentiation. These findings suggest that the Akt pathway could mediate the effects of lumican, consistent with the known critical role of Akt in osteoclast biology [30,31].

Akt is primarily activated by M-CSF [32] and thus it is quite interesting that lumican more consistently affects osteoclast lineages when both RANKL and M-CSF are present and is less effective when only M-CSF is present. Although we cannot determine the precise mechanism underlying these observations at present, there is a possibility that, despite lumican-induced Akt suppression, compensatory activation of the ERK signaling pathway may partially explain the lack of effects of lumican on proliferation and/or survival in preosteoclasts [33]. We also speculate that RANKL may increase the expression of the lumican receptor and thus the effects of lumican could be enhanced by the addition of this protein.

The RANKL and OPG secreted from osteoblast lineages are known to positively and negatively regulate osteoclastogenesis, respectively [34]. However, our study showed that the expression of *Rankl* and *Opg* was not altered in response to recombinant lumican treatment in primary osteoblasts. These results suggest that lumican directly suppresses osteoclast differentiation and bone resorption, but that its effects are not mediated by the regulation of RANKL or OPG in osteoblasts.

In summary, this study revealed that lumican, which was previously shown to have bone anabolic effects, decreases in vitro bone resorption as well as osteoclastogenesis by suppressing Akt signaling. Further animal and human studies are necessary to validate these in vitro findings and establish clinical applications.

## 4. Materials and Methods

### 4.1. Cell Culture

Primary bone marrow cells were obtained by flushing the tibias and femurs of 6-week-old ICR mice (Orient, Seongnam, Korea). The resultant cells were cultured at 37 °C in a humidified atmosphere with 5% CO_2_ in α-MEM (Welgene, Daegu, Korea) supplemented with 100 μg/mL streptomycin, 100 U/mL penicillin, and 10% FBS. After culture for 24 h, non-adherent BMMs were collected, seeded at a density of 4 × 10^4^ cells/well in 96-well culture plates, and fully differentiated into osteoclasts by culture with 30 ng/mL soluble RANKL (R&D Systems, Minneapolis, MN, USA) and 30 ng/mL M-CSF (R&D Systems) for 4 days, with culture medium replaced every 2–3 days [35]. For F-actin staining, cells were fixed with 4% paraformaldehyde for 20 min, permeabilized with 0.1% Triton X-100 for 5 min, and washed with phosphate-buffered saline (PBS). The cells were then incubated with phalloidin-FITC (Invitrogen, Carlsbad, CA, USA) for 30 min. After the cells were mounted on slides, fluorescence micrographs were acquired on an LSM 710 confocal laser scanning microscope (Carl Zeiss, Oberkochen, Germany).

Primary mouse osteoblasts were isolated by sequential collagenase digestion of calvaria obtained from newborn mice and maintained in α-MEM supplemented with 100 μg/mL streptomycin, 100 U/mL penicillin, and 10% FBS. Mature osteoclasts were generated from mouse calvaria osteoblasts by culture for 7 days in the presence of 10 mM β-glycerophosphate and 50 μg/mL ascorbic acid.

Our study was reviewed and approved by the Institutional Animal Care and Use Committee of the Asan Institute for Life Sciences (project number: 2019-12-143, approval date: 22 May 2019).

### 4.2. TRAP Stain

Adherent cells were fixed and stained using the TRAP staining kit (Sigma-Aldrich, St. Louis, MO, USA). TRAP-positive multinucleated cells containing three or more nuclei were considered to be osteoclasts. Cells were counted using a light microscope (Carl Zeiss) [36].

### 4.3. In Vitro Resorption Assay

Primary BMMs were seeded on dentine discs (IDS, Boldon, UK) at a density of 3 × 10^4^ cells/well and cultured for 10 days in the presence of 30 ng/mL RANKL and 30 ng/mL M-CSF. Cells were then completely removed by wiping with a cotton swab, and the dentine slices were stained with hematoxylin (Sigma-Aldrich) for 1 min. The area of resorbed pits was analyzed using the Image-Pro Plus software (Media Cybernetics, Silver Spring, MD, USA).

To evaluate the resorption activity of individual osteoclasts, fully differentiated osteoclasts (3 × 10^4^ cells/well in 96-well culture plates) were seeded on dentine discs and cultured for 3 days [35], and then the resorbed area was measured by the method described above.

### 4.4. Quantitative Reverse-Transcription PCR (qRT-PCR)

Total RNA was extracted using the TRIzol reagent (Invitrogen). First-strand cDNA was synthesized with the Superscript III First-Strand Synthesis System (Invitrogen) using oligo dT primers; qRT-PCR was then performed in triplicate on a Light Cycler 480 (Roche, Mannheim, Germany) using Probes Master Mix (Roche) [37]. Primers for *Trap* (Mm00475698_m1), *CatK* (Mm00484039_m1), *Ctr* (Mm00432282_m1), *Mmp9* (Mm00442991_m1), *Opg* (Mm01205928_m1), and *Rankl* (Mm00441906_m1) were obtained from Applied Biosystems (Foster City, CA, USA). The threshold cycle (Ct) value for each gene was normalized to the Ct value of 18S rRNA (Hs03928990_g1).

### 4.5. Migration Assay

The chemotaxis assay was performed in a Boyden chamber system using a Transwell with a polycarbonate membrane containing 8 μm pores (Costar, Corning, NY, USA). Cells were seeded onto the inner chamber at a density of 1.0 × 10^5^ cells per 200 μL, and then exposed to 1 nM or 10 nM lumican for 6 h. The cells on the upper membrane were completely removed by wiping with a cotton swab. Cells on the lower surface of the membrane were fixed in 4% paraformaldehyde, stained with crystal violet (Sigma-Aldrich), photographed, and counted under a dissecting microscope (Carl Zeiss).

### 4.6. Proliferation Assay

Cell proliferation was measured by monitoring 5-bromo-2′-deoxyuridine (BrdU) incorporation. Briefly, cells were seeded into a 96-well plate at 5 × 10^3^ cells per well. After 24 h, cells were incubated with BrdU for 1 day, and proliferation was assayed using the BrdU Labeling and Detection kit (Roche, Mannheim, Germany) [36]. Absorbance was read at 450 nm using a microplate reader (SPECTRAMAX 340 PC; Molecular Devices, Palo Alto, CA, USA).

### 4.7. Western Blot Analysis

Cells were lysed in RIPA buffer (50 mM Tris-HCl (pH 7.4), 1% Triton X-100, 1% sodium deoxycholate, 150 mM NaCl, 1 mM EDTA, 1 mM EGTA, 0.1% SDS, 1 mM Na_3_VO_4_, 1 mM NaF, 1 mM PMSF, and protease inhibitor cocktail). After a 30-min incubation on ice, lysates were centrifuged at 14,000× *g* rpm for 20 min at 4 °C. We used the BCA protein assay kit (Pierce Chemical, Rockford, IL, USA) to measure protein concentration. Protein samples were separated by SDS-PAGE and transferred to a polyvinylidene fluoride (PVDF) membrane followed by immunoblotting with antibodies. The following primary antibodies were purchased from Cell Signaling Technology (Danvers, MA, USA): phospho-Akt (9271), phospho-JNK (9251), phosphor-p38 (9211), phospho-ERK (9101), Akt (9272), JNK (9252), p38 (9212), and ERK (9102). Primary antibody against β-actin (A3894) was purchased from Sigma-Aldrich.

### 4.8. Apoptosis Assays

Apoptosis was assessed using the Cell Death Detection ELISA plus kit (Roche). Cells were lysed for 30 min at room temperature, followed by centrifugation at 200× *g* for 10 min. DNA fragments detected in the supernatants were used as an indicator of the extent of apoptosis. Absorbance was read at 405 nm on a microplate reader (SPECTRAmax 340 PC).

Apoptosis was also measured by terminal deoxynucleotidyl transferase-mediated dUTP-biotin nick end-labeling (TUNEL) assay using the TACS Blue label kit (4811-30-K, R&D Systems). Briefly, cells were sequentially treated with terminal deoxynucleotidyl transferase, biotinylated nucleotides, and horseradish peroxidase-conjugated streptavidin, and then developed in diaminobenzidine solution. Cells were counterstained with methyl green. Apoptotic cells were counted in four randomly selected visual fields for each sample.

### 4.9. Viability Assay

Cell viability was measured using the Cell Counting Kit-8 (CCK-8; Dojindo, Kumamoto, Japan) according to the manufacturer’s instructions. Briefly, 10 μL of WST-8 dye [2-(2-methoxy-4-nitrophenyl)-3-(4-nitrophenyl)-5-(2,4-disulfophenyl)-2H-tetrazolium, monosodium salt] was added to each well of a 96-well plate and then the plates were incubated for 1 h before being subjected to evaluation at 450 nm using a microplate reader (SPECTRAmax 340PC).

### 4.10. Statistical Analyses

All data are expressed as means ± standard error of the mean (SEM) from at least three independent experiments with triplicate measurements, unless otherwise specified. The significance of differences among three or more groups was tested by ANOVA with post hoc analysis with Tukey’s HSD test, and differences between two groups was assessed using the Mann–Whitney U test [38]. All statistical analyses were performed using SPSS version 18.0 (SPSS, Inc., Chicago, IL, USA). *p* < 0.05 was considered statistically significant.

## Data Availability

The data used to support the findings of this study are available from the corresponding authors upon request.

## Supplementary Materials

The supplementary materials are available online at https://www.mdpi.com/article/10.3390/ijms22094717/s1.

## References

[1] Schaefer L., Iozzo R.V. (2008). Biological functions of the small leucine-rich proteoglycans: From genetics to signal transduction. J. Biol. Chem..

[2] Iozzo R.V. (1998). Matrix proteoglycans: From molecular design to cellular function. Annu. Rev. Biochem..

[3] McEwan P.A., Scott P.G., Bishop P.N., Bella J. (2006). Structural correlations in the family of small leucine-rich repeat proteins and proteoglycans. J. Struct. Biol..

[4] Merline R., Schaefer R.M., Schaefer L. (2009). The matricellular functions of small leucine-rich proteoglycans (SLRPs). J. Cell Commun. Signal..

[5] Zappia J., Joiret M., Sanchez C., Lambert C., Geris L., Muller M., Henrotin Y. (2020). From Translation to Protein Degradation as Mechanisms for Regulating Biological Functions: A Review on the SLRP Family in Skeletal Tissues. Biomolecules.

[6] Nikitovic D., Aggelidakis J., Young M.F., Iozzo R.V., Karamanos N.K., Tzanakakis G.N. (2012). The biology of small leucine-rich proteoglycans in bone pathophysiology. J. Biol. Chem..

[7] Young M.F., Bi Y., Ameye L., Xu T., Wadhwa S., Heegaard A., Kilts T., Chen X.D. (2006). Small leucine-rich proteoglycans in the aging skeleton. J. Musculoskelet. Neuronal Interact..

[8] Xu T., Bianco P., Fisher L.W., Longenecker G., Smith E., Goldstein S., Bonadio J., Boskey A., Heegaard A.M., Sommer B. (1998). Targeted disruption of the biglycan gene leads to an osteoporosis-like phenotype in mice. Nat. Genet..

[9] Bi Y., Nielsen K.L., Kilts T.M., Yoon A.M.A.K., Wimer H.F., Greenfield E.M., Heegaard A.M., Young M.F. (2006). Biglycan deficiency increases osteoclast differentiation and activity due to defective osteoblasts. Bone.

[10] Nuka S., Zhou W., Henry S.P., Gendron C.M., Schultz J.B., Shinomura T., Johnson J., Wang Y., Keene D.R., Ramirez-Solis R. (2010). Phenotypic characterization of epiphycan-deficient and epiphycan/biglycan double-deficient mice. Osteoarthr. Cartil..

[11] Nikitovic D., Katonis P., Tsatsakis A., Karamanos N.K., Tzanakakis G.N. (2008). Lumican, a small leucine-rich proteoglycan. IUBMB Life.

[12] Nikitovic D., Berdiaki A., Zafiropoulos A., Katonis P., Tsatsakis A., Karamanos N.K., Tzanakakis G.N. (2008). Lumican expression is positively correlated with the differentiation and negatively with the growth of human osteosarcoma cells. FEBS J..

[13] D’Onofrio M.F., Brezillon S., Baranek T., Perreau C., Roughley P.J., Maquart F.X., Wegrowski Y. (2008). Identification of beta1 integrin as mediator of melanoma cell adhesion to lumican. Biochem. Biophys. Res. Commun..

[14] Lee J.Y., Park S.J., Kim D.A., Lee S.H., Koh J.M., Kim B.J. (2020). Muscle-Derived Lumican Stimulates Bone Formation via Integrin α2β1 and the Downstream ERK Signal. Front. Cell Dev. Biol..

[15] Boyce B.F. (2013). Advances in osteoclast biology reveal potential new drug targets and new roles for osteoclasts. J. Bone Miner. Res..

[16] Yim M. (2020). The Role of Toll-Like Receptors in Osteoclastogenesis. J. Bone Metab..

[17] Asagiri M., Takayanagi H. (2007). The molecular understanding of osteoclast differentiation. Bone.

[18] Matsumoto T., Nagase Y., Hirose J., Tokuyama N., Yasui T., Kadono Y., Ueki K., Kadowaki T., Nakamura K., Tanaka S. (2013). Regulation of bone resorption and sealing zone formation in osteoclasts occurs through protein kinase B-mediated microtubule stabilization. J. Bone Miner. Res..

[19] Sugatani T., Hruska K.A. (2005). Akt1/Akt2 and mammalian target of rapamycin/Bim play critical roles in osteoclast differentiation and survival, respectively, whereas Akt is dispensable for cell survival in isolated osteoclast precursors. J. Biol. Chem..

[20] Vergadi E., Ieronymaki E., Lyroni K., Vaporidi K., Tsatsanis C. (2017). Akt Signaling Pathway in Macrophage Activation and M1/M2 Polarization. J. Immunol..

[21] Yang D., Wan Y. (2019). Molecular determinants for the polarization of macrophage and osteoclast. Semin. Immunopathol..

[22] Kim B.J., Koh J.M. (2019). Coupling factors involved in preserving bone balance. Cell. Mol. Life Sci..

[23] Rachner T.D., Khosla S., Hofbauer L.C. (2011). Osteoporosis: Now and the future. Lancet.

[24] Reyes C., Hitz M., Prieto-Alhambra D., Abrahamsen B. (2016). Risks and Benefits of Bisphosphonate Therapies. J. Cell. Biochem..

[25] Min Y.K. (2015). Update on denosumab treatment in postmenopausal women with osteoporosis. Endocrinol. Metab..

[26] Seomun Y., Joo C.-K. (2008). Lumican induces human corneal epithelial cell migration and integrin expression via ERK 1/2 signaling. Biochem. Biophys. Res. Commun..

[27] Brézillon S., Pietraszek K., Maquart F.-X., Wegrowski Y. (2013). Lumican effects in the control of tumour progression and their links with metalloproteinases and integrins. FEBS J..

[28] Duong L.T., Lakkakorpi P., Nakamura I., Rodan G.A. (2000). Integrins and signaling in osteoclast function. Matrix Biol. J. Int. Soc. Matrix Biol..

[29] Yasuda H., Shima N., Nakagawa N., Yamaguchi K., Kinosaki M., Mochizuki S., Tomoyasu A., Yano K., Goto M., Murakami A. (1998). Osteoclast differentiation factor is a ligand for osteoprotegerin/osteoclastogenesis-inhibitory factor and is identical to TRANCE/RANKL. Proc. Natl. Acad. Sci. USA.

[30] Kim J.H., Kim N. (2016). Signaling Pathways in Osteoclast Differentiation. Chonnam. Med.J..

[31] Moon J.B., Kim J.H., Kim K., Youn B.U., Ko A., Lee S.Y., Kim N. (2012). Akt induces osteoclast differentiation through regulating the GSK3β/NFATc1 signaling cascade. J. Immunol..

[32] Golden L.H., Insogna K.L. (2004). The expanding role of PI3-kinase in bone. Bone.

[33] Lee K., Seo I., Choi M.H., Jeong D. (2018). Roles of Mitogen-Activated Protein Kinases in Osteoclast Biology. Int. J. Mol. Sci..

[34] Udagawa N., Koide M., Nakamura M., Nakamichi Y., Yamashita T., Uehara S., Kobayashi Y., Furuya Y., Yasuda H., Fukuda C. (2021). Osteoclast differentiation by RANKL and OPG signaling pathways. J. Bone Miner. Metab..

[35] Park S.J., Lee J.Y., Lee S.H., Koh J.M., Kim B.J. (2019). SLIT2 inhibits osteoclastogenesis and bone resorption by suppression of Cdc42 activity. Biochem. Biophys. Res. Commun..

[36] Kim B.J., Lee Y.S., Lee S.Y., Baek W.Y., Choi Y.J., Moon S.A., Lee S.H., Kim J.E., Chang E.J., Kim E.Y. (2018). Osteoclast-secreted SLIT3 coordinates bone resorption and formation. J. Clin. Investig..

[37] Kim B.J., Lee J.Y., Park S.J., Lee S.H., Kim S.J., Yoo H.J., Rivera De Pena S.I., McGee-Lawrence M., Isales C.M., Koh J.M. (2019). Elevated ceramides 18:0 and 24:1 with aging are associated with hip fracture risk through increased bone resorption. Aging.

[38] Kim D.A., Park S.J., Lee J.Y., Kim J.H., Lee S., Lee E., Jang I.Y., Jung H.W., Park J.H., Kim B.J. (2021). Effect of CCL11 on In Vitro Myogenesis and Its Clinical Relevance for Sarcopenia in Older Adults. Endocrinol. Metab..

