# Supplementary material for: Lumican Inhibits Osteoclastogenesis and Bone Resorption by Suppressing Akt Activity
Source: Int J Mol Sci. 2021 Apr 29;22(9):4717. doi: 10.3390/ijms22094717 (PMC8124849; doi:10.3390/ijms22094717)
Supplement: Supplementary file 1 [file ijms-22-04717-s001.zip › ijms-1149430-supplementary.pdf]

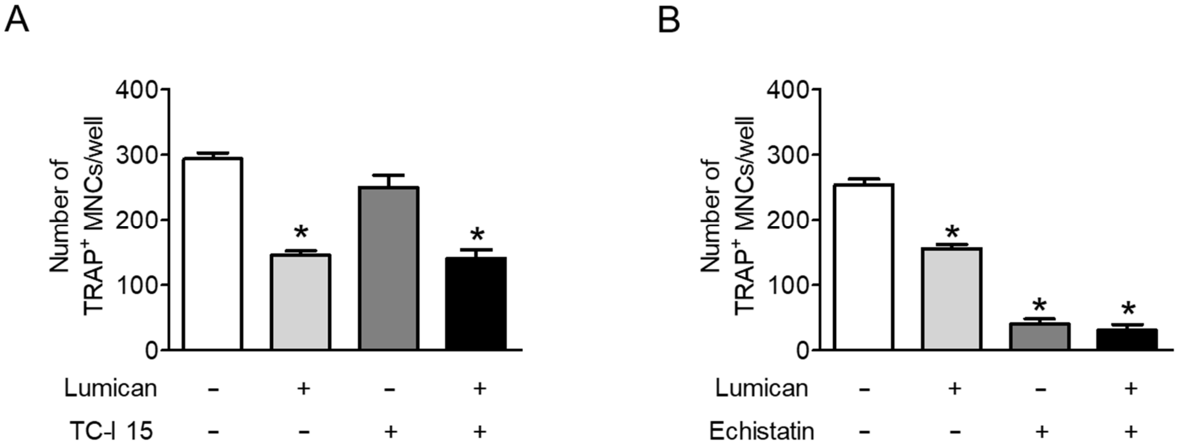

Supplemental Figure 1. The effect of lumican on osteoclastogenesis is not reversed by the inhibitors of integrin $\alpha2\beta1$ or $\alpha V\beta3$. (A and B) Primary mouse BMMs were treated with 30 ng/mL RANKL, 30 ng/mL M-CSF, 10 nM lumican, and (A) 1 μM TC-I 15 (integrin $\alpha2\beta1$ inhibitor) or (B) 10 nM echistatin (integrin $\alpha V\beta3$ inhibitor) for 4 days. The cells were then stained with TRAP, and the number of TRAP-positive multinucleated cells (MNCs) (≥ 3 nuclei/cell) was determined to assess osteoclast differentiation (n = 3). Data are presented as the mean ± SEM. **P* < 0.05 vs. untreated control.
